# Structure Prediction and Expression of Modified rCTLA4-Ig as a Blocker for B7 Molecules

**DOI:** 10.22037/ijpr.2020.112959.14040

**Published:** 2020

**Authors:** Hossein Mahdizadeh, Jafar Salimian, Zahra Noormohammadi, Jafar Amani, Raheleh Halabian, Yunes Panahi

**Affiliations:** a *Department of Biology, Science and Research branch, Islamic Azad University, Tehran, Iran. *; b *Chemical Injuries Research Center, Systems Biology and Poisonings Institute, Baqiyatallah University of Medical Sciences, Tehran, Iran. *; c *Applied Microbiology Research Center, Systems Biology and Poisonings Institute, Baqiyatallah University of Medical Sciences, Tehran, Iran.*

**Keywords:** CTLA4-Ig, Belatacept, Bioinformatics analysis, Rrecombinant protein, Drug design

## Abstract

CTLA4-Ig (Abatacept) has been produced to suppress immune response by inhibition of T cells functions in autoimmune disease. A new drug, which is called belatacept, has recently been recently developed that is more efficient. The development has been occurred by two substitutions (A29Y, L104E) in the extracellular domain of CTLA4. In the present study, the bioinformatics analysis was used in order to make a new structure that has a better function in comparison with belatacept. Firstly, eight different structures were designed. Thereafter, the secondary and 3D structures, mRNA structure, docking of chimeric proteins with CD80/CD86, antigenicity and affinity of designed chimeric molecules were predicted. Based on the criteria, a new candidate molecule was selected and its gene synthesized. The gene was cloned and expressed in *E. coli* BL21 (DE3) successfully. The purified rCTLA4-Ig was analyzed by SDS-PAGE, western blotting, and ELISA. Circular dichroism analysis (CD analysis) was used for characterization of the rCTLA4-Ig. Affinity of rCTLA4-Ig was also evaluated by the flow cytometry method. Finally, its biological activity was determined by T cell inhibition test. The results showed rCTLA4-Ig and the belatacept protein have some similarities in structure and function. In addition, rCTLA4-Ig was able to bind CD80/CD86 and inhibit T cell function. Although flow cytomery results showed that the standard protein (CTLA4-Ig), represented better affinity than rCTLA4-Ig, the recombinant protein was able to inhibit T cell proliferation as well as CTLA4-Ig.

## Introduction

T cells Activation needs two signals, which are provided by antigen presenting cells (APC). The initial signal is activated by binding antigenic peptides, which bound to MHC class I or II molecules that are expressed by APCs to TCR on T cells. The second signal can be provided by costimulatory molecules. One of these molecules is CD28, which is the best characterized ligand. It is expressed by T cells, which binds to CD80 (B7.1) or CD86 (B7.2) on APCs (1). Some family members of CD28 receptors have inhibitory roles. CTLA4 (CD152) is one of these family members, which are expressed on the surface of activated T cells. Although, it is expressed at low levels by T cells, it has a greater affinity to CD80 and CD86 in comparison with CD28 (2-4). The ability of CTLA4 to inhibit T cells and limit the immune response has raised this molecule as a target for treating autoimmune diseases (5). The fusion protein was constructed of extra cellular domain of CTLA4 and FC fragment of human IgG1 (Abatacept) interferes with CD28/B7 interaction and inhibits the T cell activation. Abatacept has been made to treat rheumatoid arthritis and reduces joint damage. It also has great potential to treat other autoimmune diseases such as Systemic lupus erythematic (SLE) (6, 7). Recently, belatacept which has two amino acid substitutions (A29Y, L104E) in CTLA4 binding motif, with improved avidity to CD80 and CD86 has been approved for the therapy of transplant rejection, especially in renal transplants (8, 9). Xu Z. *et al*. (2012) reported that a residue substitution in porcine and human CTLA-4 (L to M substitution at position 97) changes affinity of CTLA4 to CD80 and CD86 (10, 11).

The present study sought to produce a novel recombinant protein which have more stability and increase its binding affinity. Some chimeric proteins were designed based on different substitutions in CTLA4 fragment, and the stabilizer fragment (FC) was exchanged with 3DHSA (domain III of Human Serum Albumin). Then, structural and functional properties of designed chimeric proteins were compared with belatacept by the bioinformatics. After the selection of the best structure, the gene of selected protein was synthesized and it was produced as rCTLA4-Ig. Finally, the structure and function of protein were evaluated. 

## Experimental

*Bioinformatics analysis*

*design of chimeric protein*

Related sequence of extracellular domain of CTLA4 (Accession number: P16410), FC fragment of IgG1 (Accession number: 075015) and the third domain of human serum albumin (3DHSA) (Accession number: P02768) were adopted from Uniprot database. Abatacept and Belatacept sequences were found out from DRUG BANK (http://www.drugbank.ca/). Two kinds of chimeric proteins were designed. These designs were performed to achieve a structure with two characteristics, more stability and increase its affinity binding. The first one was made by joining the C terminal of extra domain of CTLA4 to N terminal of third domain of human serum albumin (3DHSA), which acts as a stabilizer. The fusion was done either by a flexible or rigid linker separately. The second kind of chimeric protein was designed by fusing the C terminal of extra domain of CTLA4 to N terminal of FC fragment of human IgG1 as a stabilizer via a linker. Also some amino acid residues of CTLA4 were substituted so as to evaluate their affects on affinity of chimeric protein to CD80 and CD86.

*Constructs analysis*

Physical and chemical parameters of constructs including the molecular weight, theoretical pI, amino acid composition, atomic composition, extinction coefficient, estimated half-life, instability index, aliphatic index, and grand average of hydropathicity (GRAVY) were analyzed by Expasy’s protparam (12). The mRNA secondary structures was predicted by the mfold server (http://mfold.rna.albany.edu/) (13, 14). Garnier-Osguthorpe-Robson (GOR) IV secondary structure prediction method was performed to analyze secondary structure of designed constructs (15, 16) and proportion of random coils, extended strands, and alpha helices of chimeric proteins were determined. The iterative threading assembly reﬁnement (I-TASSER) (http://zhanglab.ccmb.med.umich.edu/I-TASSER/) was used for tertiary structure prediction (17-19). Accelrys Discovery Studio 2.5 software was used to visualize the modeled tertiary structures. The structures were evaluated by Rampage, proSA, and verify3D servers (20). The interaction of the designed chimeric proteins with their ligands was analyzed by Zdock server (http://zdock.umassmed.edu/) (21).The binding affinity of chimeric proteins to CD80 and CD86 was predicted by PPA-Pred server (http://www.iitm.ac.in/bioinfo/PPA-Pred/) (22, 23). The antigenicity of chimeric proteins was evaluated by VaxiJen server (http://www.ddg-pharmfac.net/vaxijen/scripts/VaxiJen_scripts/VaxiJen3.pl) (24-26).

*Experimental Analysis*

*production of recombinant chimeric protein (rCTLA4-Ig)*

Based on computational study, the sequence of the selected chimeric protein was turned back into DNA. The gene sequence of structure 6 (rCTLA4-Ig) was optimized and synthesized for expression in *E.coli* host (Biomatic company, Canada). Forward and reverse primers containing *HindIII* and *XhoI* (Fermentas, Canada) restriction sites were designed and after performing PCR with *Pfu *DNA polymerase (Thermofisher, USA), structure 6 gene was directly cloned with the same restriction sites in the *pET28a* expression vector (Invitrogen, USA). the clones were confirmed by PCR and restriction enzymatic digestion. Sequencing was performed to confirm the homology of the cloned DNA fragment with the expected rCTLA4-Ig sequence.

 Then, the recombinant vector was transformed into *E.coli* strain *BL21* (*DE3*) (Invitrogen, USA). The recombinant bacterial cells were inoculated into 10ml LB broth supplemented with 50μg/ml kanamycin and cultured overnight at 37 °C with shaking at 150 rpm. The overnight culture was sub cultured and grown in 50mL medium until the OD_600_ reached 0.7. Then, 1mM IPTG was added to induce protein expression and incubation was continued for 6 h. Bacterial pellets were dissolved in buffer B (Urea 8M, NaH_2_PO_4 _100 mM, Tris HCl 10mM) and the suspension was sonicated. The molecular weight of rCTLA4-Ig was determined by 12% sodium dodecyl sulfate-polyacrylamide gel electrophoresis (SDS-PAGE).

*Purification of rCTLA4-Ig*

The His-tagged chimeric protein (rCTLA4-Ig) was purified by Ni-NTA affinity chromatography (QIAGEN, USA). On-column refolding and Purification were performed. After collecting the pellets, binding buffer (Urea 8M, NaH_2_PO_4 _100 mM, Tris HCl 10 mM) was added and was shaken at 50 rpm for 30 min at room temperature. Cell lysate was centrifuged at 10000g for 30 min. The supernatant was mixed to Ni-NTA resins and was incubated and shaken for 2 h at room temperature. The column was packed. Then, denaturation wash buffer (100mM NaH_2_PO4, 10 mM Tris HCl, 8M urea), renaturation wash buffer 1-6 (50mM NaH_2_PO4, 300 mM NaCl, Urea 8-6-4-2-1 and 0 M), native wash buffer (50mM NaH_2_PO4, 300 mM NaCl, 20mM imidazole pH: 8), and elution buffer (50mM NaH_2_PO4, 300 mM NaCl, 250mM imidazole pH = 8) were respectively poured onto column. Elution buffer was finally collected and purified rCTLA4-Ig was stored at -20°C. Protein concentration was measured by Bradford method (27). Firstly, the standard curve was determined based on BSA protein. Then, the rCTLA4-Ig sample concentration was assessed.

*Evaluation of rCTLA4-Ig using ELISA test*

Initially, serial concentrations (0.3-5μg/100µL) of rCTLA4-Ig mixed with coating buffer (Na_2_Co_3_, NaHCo_3_, 0.05 M, pH 9.8) were poured in ELISA plate (Biobasic, Canada) and incubated overnight at 4 °C. The same serial dilution of BSA (Biobasic, Canada) was also used as a control. The plates were washed by PBST (PBS containing 0.05% Tween). Then, they were blocked with blocking buffer (PBST with 3% skim milk) and incubated for 1 h at 37 °C. After being washed with PBST, anti-human IgG-horseradish peroxidase antibody (1/10000) (Sigma Aldrich, USA) was added and incubated for 2 h at 37 °C. The plates were washed with PBST again and TMB Substrate was poured and incubated for 20 min at 37 °C. Stop solution was eventually added. The result was analyzed by ELISA Reader (Sigma, USA). The ELISA test was repeated three times**.**

*Confirmation of rCTL4-Ig expression by Western Blotting*

 After running rCTL4-Ig by SDS-PAGE, a semidry blotting was performed to transfer protein to polyvinylidene fluoride (PVDF) membrane (Roche, Germany). The membrane was incubated in blocking buffer (3% skim milk in PBST pH = 7.2) overnight at 4 °C. After being washed by PBST, the membrane was put in anti-human IgG-horseradish peroxidase antibody (1:10,000) (Sigma Aldrich, USA) for 2 h at room temperature with shaking at 50 rpm. The membrane was washed by PBST again. Then, it was stained with DAB (3,3’Diaminobanzidene) (Sigma, USA) to reveal protein bands. The reaction was stopped by rinsing the membrane with distilled water.

*Circular dichroism (CD) analysis*

Circular dichroism was performed using JASCO J-810 spectropolarimeter (Applied Photophysics, UK) to compare the second structure of the rCTLA4-Ig with predicted structure. The apparatus was set with a 1mm path length and measurement range 240 - 180 nm at 25 °C. Protein concentration which was dissolved in phosphate saline buffer was 0.2 mg / mL.

*Flow cytometry analysis*

The affinity of rCTLA4-Ig to B7 receptors which are expressed by *Raji* cells )Pastur, Iran) was evaluated by flow cytometry. *HEK-293* cells )Pastur, Iran) that do not express B7 receptors were used as negative control. Briefly, 100,000 cells were poured into each tube and washed with cold PBS. Blocking was performed by incubating the cells with 100 μL PBS containing 2% FBS for 45 min at 4 °C. The mouse anti-human CD80-PE (BD, USA) was added to confirm the expression of the CD80 receptor on *Raji* cells. The analysis was performed by the BD FACSCalibur flow cytometery device (BD, USA) after incubating for 45 min at 4 °C. Then, serial concentrations of rCTLA4-Ig (10μg/mL, 1μg/mL, 0.1μg/mL, and 0.01μg/mL) were added to the *Raji* cells and were incubated for 45 min at 4 °C. Then, monoclonal anti-human IgG1-FITC (Sigma Aldrich, USA) was added to the cells and incubated for 45 min at 4 °C. The analysis was performed by the BD FACSCalibur flow cytometery device. The standard CTLA4-Ig (Adipogen( was used as positive control. This product has been made in *CHO* cells according to company data sheet. Flowjo7 software was used to analyze acquired data. The experiments were repeated three times**.**

*T lymphocyte inhibition by rCTLA4-Ig*

At first, 200 μL of a mixture, consisting of CtxB (B subunit of Chlora toxin), sterile saline buffer, and adjuvant, was injected to five BALB/c mice (Pastur, Iran). The injections were done subcutaneously on four occasions with 2 weeks intervals between them. For lymphocyte proliferation, splenocytes (50,000 cells/ well) of immunized mice were grown in 96 wells plate. 4 μg/mL concanavalin A (Sigma, USA) and 10 μg/mL of CtxB antigen were added to all wells. Then, different concentrations (20 μg/mL, 10 μg/mL, 5 μg/mL, and 2.5 μg/mL) of rCTLA4-Ig were added and the plate was incubated for 48 h at 37 °C. rCTLA4-Ig was not used in the control well. Eventually, the viability of the treated cells was measured by MTT assay. The experiments were repeated three times**.**

## Results

*Design of chimeric proteins construct and optimization*

**Figure 1:**
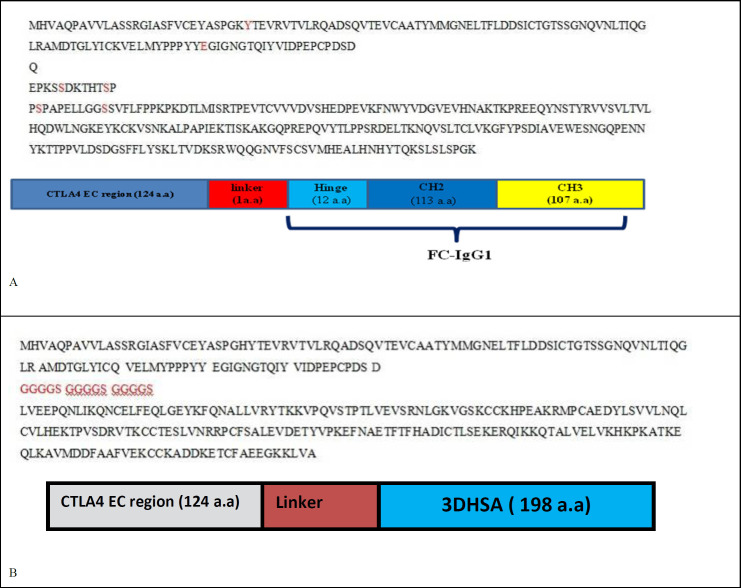
Gene sequences and Overview diagrams of **A)** Abatacept and belatacept **B)** structures 1 and 2

We have designed abatacept and belatacept constructs and eight other structures according to Table 1. Gene sequences and overview diagrams of designed chimeric proteins are represented in Figure 1.

*Physicochemical properties of the chimeric proteins*

Physicochemical properties of the chimeric proteins and the computed parameters, including molecular weight, theoretical pI, estimated half-life, instability index, aliphatic index, and grand average of hydropathicity (GRAVY) were obtained by Expasy’s protparam (Table 2). 

*Secondary and 3D structure prediction*

**Figure 2:**
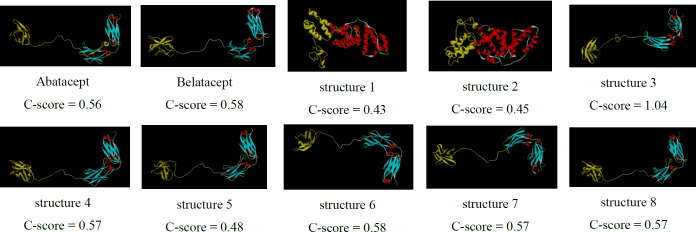
3D structure prediction of the chimeric proteins by I-TASSER server. Structure 3-8 show two distinct sections separated by linkers as well as belatacept. But, structures 1, 2 represent different conformation

According to GORIV prediction, proportion of random coils, extended strands, and alpha helices of chimeric proteins were calculated (Table 3). All of the chimeric proteins were almost identical in the second structure except for structures 1 and 2, which had higher alpha helix content. Figure 2 shows predicted 3D structure of the chimeric proteins (predicted by I-TASSER server). 

Structure 3 had the lowest confidence score (C-score = -1.04) comparing with the other constructs. C-score is typically in the range of [−5, 2] and higher values of c-score signify a model with a high confidence.

*Evaluation of structural modeling*

**Figure 3:**
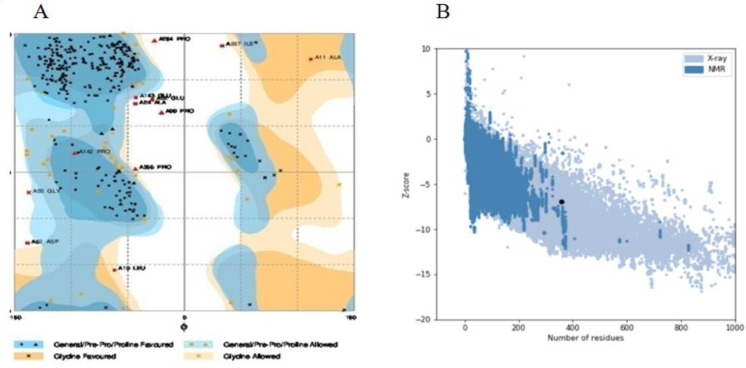
Validation of structure 6 by (A) Ramachandran plot using RAMPAGE server, 85.9% of amino acid residues were incorporated in favored region, 10.7% of the residues were in allowed regions and only 3.4% of residues were in outlier region. (B) ProSA server, structure 6 score (black dot) was intside a range characteristic for native proteins

The structural stability of the chimeric proteins was confirmed by Ramachandran plot. Number of residues in favored region, number of residues in the allowed region, and number of residues in outlier region are shown in table 4. In all of the chimeric proteins, most of the residues are in favored region. Therefore, they are stable. Ramachandran plot of structure 6 is depicted in figure 3A. In addition, overall quality score for structure 6 was calculated by ProSA server and, its score was in a range characteristic for native proteins (Figure 3B). Verify3D analyses of structures also represented that just structure 6 and 8 more than 80% of the amino acid residues have scored > = 0.2 in the 3D/1D profile. On the other hand, Structure 6 with 87.67% of residues had better result than structure 8 with 83.19% of residues. So the structure 6 was selected. 

*mRNA structure properties*

**Figure 4:**
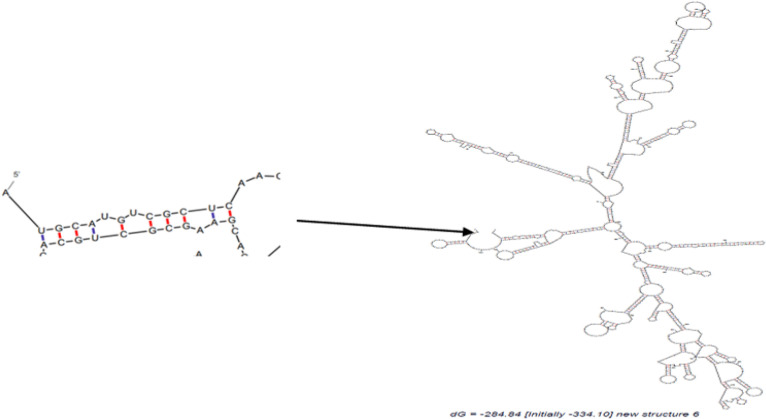
The optimal secondary structure of Structure 6 mRNA using mfold. Predicted structure has no long stable hairpin and pseudo knot at the 5′ end of mRNA

mRNA Secondary structures of chimeric proteins were predicted by mfold. Only mRNA secondary structure of Structure 6 is depicted in Figure 4. Predicted structures had no long stable hairpin and pseudo knot at the 5′ end of mRNA. The minimum free energy of the secondary structures was also predicted. Thermodynamic details related to 5’ end of mRNAs were obtained by mfold tool (Table 5). The messenger RNA secondary structure of the chimeric gene was analyzed with these parameters: Linear RNA folding at 5%, window: 12, max folds: 50. All structural elements obtained in this analysis have revealed folding of the RNA construct at 37 °C with Initial ΔG ranging from -321.60 to -377.30 kcal/mol.

*Docking of chimeric proteins with CD80 and CD86*

**Figure 5:**
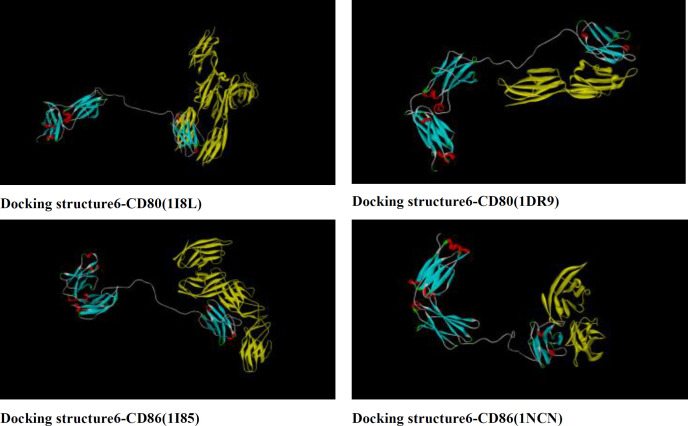
Docking of structure 6 with CD80 (1I8L and 1DR9) and CD86 (1I85 and 1NCN) using Zdock server.CD80 and CD86 receptors bind properly to functional domain of rCTLA4-Ig

Docking of structure 6 with CD80 and CD86 ligands were predicted and compared with standard protein by Zdock server. Zdock server is fast fourier transform based protein docking program authored and maintained by Zhiping weng’s lab (ZLAB) at the university of massachusetts medical school. Zdock searches all possible binding modes in the translational and rotational space between the two proteins and evaluates each pose using an energy-based scoring function. PDB formats of CD86 (1I85 and 1NCN) and CD80 (1I8L and 1DR9) ligands were obtained from uniprot database. (http://www.uniprot.org/uniprot/P33681). Based on docking illustrated in figure 5**, **B7 receptors bound to functional domain of rCTLA4-Ig same as CTLA4-Ig**.**

*Affinity prediction of chimeric proteins with CD80 and CD86*

Affinity of chimeric proteins with CD80 and CD86 were predicted by PPA-Pred server. As it is shown in table 6, the best affinity is related to structure 2 with-15.65 kcal /mol for CD80 and-14.03 kcal/mol for CD86. Predicted value of Kd (dissociation constant) of structure 2 is 9.76e-12 M for CD80 and 1.55e-10 M for CD86. Affinity of the other structures was approximately similar to belatacept.

*Antigenicity evaluation*

To predict antigenicity index of chimeric proteins, VaxiJen server was used with these parameters: model selected = tumour and threshold for this model = 0.5. Structure 1, 2, 3 with overall prediction for antigen respectively, 0.6558, 0.6105 and 0.5099 were probable antigen. Other structures are probable non-antigen as it is shown in Table7. 

*Production of rCTLA4-Ig*

**Figure 6:**
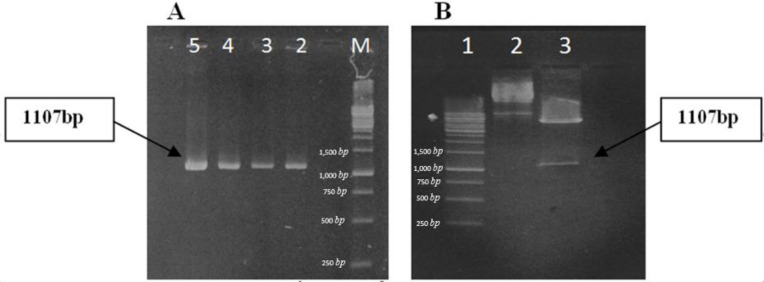
Confirmation of cloning by PCR and enzyme digestion (**A) **PCR reaction: M, DNA ladder (1kb); lanes 2-5, PCR product. Correct bands are revealed on gel. (**B)** Enzyme digestion of recombinant plasmid: lane 1, ladder; lane 2, uncut plasmids; lane 3, digested plasmids; enzyme digestion is accomplished. An 1107bp fragment is produced

 Cloning of chimeric gene was confirmed by PCR reaction and *HindIII* and *Xho*1 restriction enzyme digestion. As it is depicted in figure 6, PCR result showed that reaction amplifies a correct 1107 bp fragment. A same fragment was also produced after enzyme digestion. On the other hand, the homology of the cloned DNA fragment with the expected rCTLA4-Ig sequence was confirmed by sequencing. Cultured *E.coli (DE3)* cells were induced by *IPTG* to produce recombinant protein. Optimum condition for expression of rCTLA4-IG was carried out after 6 h induction at 37 °C. 

*Purification of rCTLA4-Ig*

**Figure 7:**
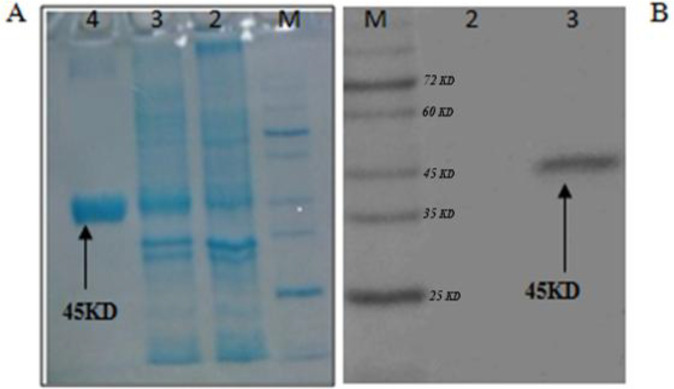
(A) Running of rCTLA4-Ig on SDS-PAGE.: M, protein ladder; lane 2, bacterial extraction before induction; lane 3, bacterial extraction after induction with IPTG; lane 4, purification of rCTLA4-Ig by Ni-NTA column. (B)Western blotting confirms the predicted molecular weight. M, prestained protein ladder; lane2, Negative control; lane 3, rCTLA4-Ig protein band

The bacterium containing the recombinant plasmid was cultured in large scale, then, induced by 1mM IPTG. Purification of rCTLA4-Ig was accomplished under denaturing conditions. After cell collection, an on-column refolding and purification protocol was operated for rCTLA4-Ig purification (Figure 7A). The 6His-tag part in N terminal of protein strongly bound to Ni-NTA column. Thus, the protein was properly purified in large scale. Purified protein concentration was measured by Bradford method and its concentration based on the volume of culture was about 10 µg /mL*.* The binding characteristics of purified rCTLA4-Ig were analyzed by Western blotting (Figure ig.7B).

*rCTLA4-Ig Confirmation by ELISA*

**Figure 8:**
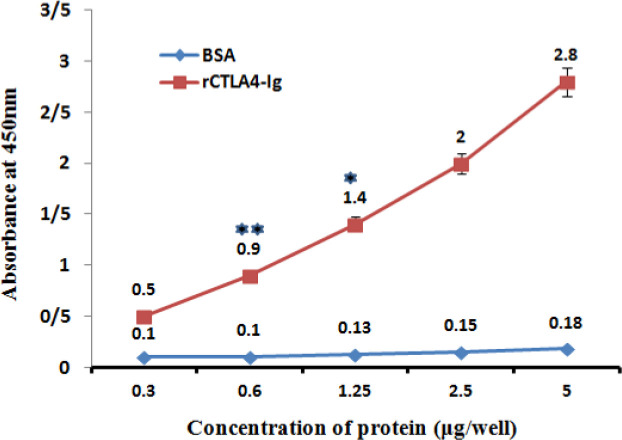
Interaction of Chimeric rCTLA4-Ig with Anti-IgG antibody. The absorbance increased in higher concentration of rCTLA4-Ig logically (red line). whereas, BSA absorbance stayed in baseline level*.* Value significantly different from BSA only at *P < 0.05 or **P < 0.01

The rCTLA4-Ig was confirmed by ELISA test using anti human IgG antibody. Anti-IgG antibody bound to FC fragment of rCTLA4-Ig successfully. There was a direct relation between rCTLA4-Ig concentration and absorbance (OD). The binding of anti- human IgG antibody to BSA was at the base line level in negative control. For statistical analysis the test was repeated three times and all of tests had almost similar results. The average of quantities is shown in Figure 8.

*Circular dichroism*

The CD spectrum of structure 6 was measured at pH 7.4 and room temperature. Secondary structure CDNN software was used to analyze CD spectra. As it is mentioned in table 8, CD spectra analysis and predicted data are almost similar. 

*Flow cytometry analysis*

*Raji* cells were stained by the mouse anti-human CD80-PE antibody. The cells that were not incubated with antibody were used as a baseline of fluorescent intensity. A shift in the fluorescence spectra demonstrated that antibody bound to CD80 receptors on cell surface. Fluorescent intensity more than 90% demonstrated high expression level of the CD80 receptor on surface of *Raji* cells. *HEK-293* cells which do not have any CD80 receptors on their surface were selected for negative control. A lack of fluorescence shift Showed that antibodies did not bind to negative control cells (*HEK-293*).

The standard protein which consists of the extracellular domain of human CD152 [CTLA-4] (aa 37-160) and FC region of human IgG1 was produced in *CHO* cells. According to biological activity on data sheet, it binds both CD80 (B7-1) and CD86 (B7-2) with high affinity and inhibits CD28 signaling competitively and kills the target cell completely.

**Figure 9:**
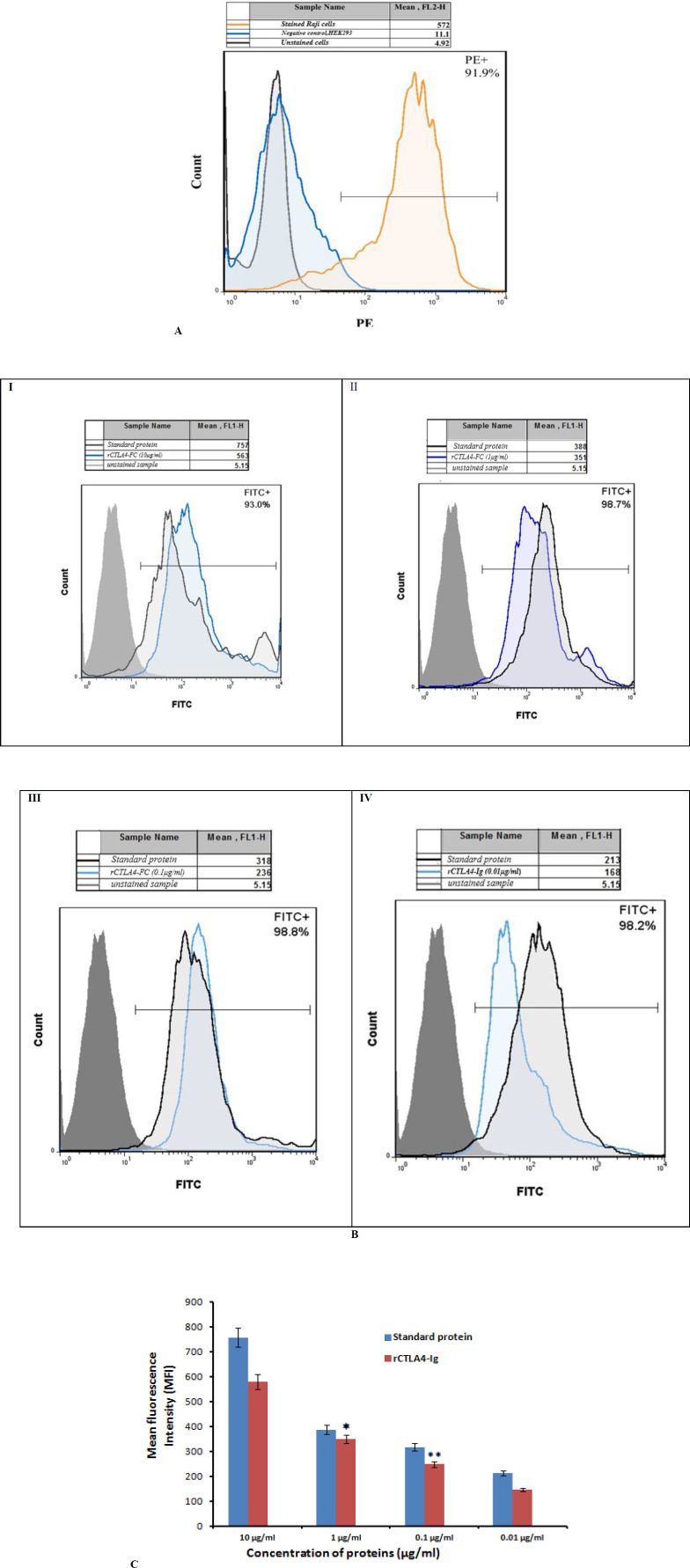
**(A).** The fluorescent intensity of mouse anti-human CD80-PE antibody on the Raji cells. Raji unstained cells that were not incubated with antibodies were used as a baseline of fluorescent intensity. (Grey graph) The orange graph demonstrates stain of CD80 on Raji cells. A lack of fluorescence shift demonstrated that antibodies did not bind nonspecifically to negative control, HEK293 (blue graph) (B) Comparison of fluorescent intensity of monoclonal anti-human IgG1-FITC antibody bound to rCTLA4-Ig at different concentrations: I) 10 μg/mL, II) 1μg/mL, III**) **0.1 μg/mL, IV) 0.01 μg/mL. In all graphs, the right-shifted blue graphs represent bound rCTLA4-Ig detected with anti-human IgG1-FITC antibodies. The right-shifted black graphs demonstrate bound standard protein as positive control. The gray graphs indicate the background fluorescence (unstained sample) in comparison. The tables above all graphs represent mean fluorescent intensity which was analyzesd by flowjo7 software. C) The bar graph of mean fluorescent intensity (MFI) of rCTLA4-Ig and standard protein (CTLA4-Ig): The MFI logically reduced by decreasing rCTLA4-Ig concentrations due to stronger affinity of rCTLA4-Ig to B7 receptors in comparison with standard protein. Value significantly different from standard protein only at *P < 0.05 or **P < 0.01

Fluorescent intensity of monoclonal anti-human IgG1-FITC antibody in all concentrations of standard protein and rCTLA4-Ig was also above 90% (Figure 9B), whereas the mean fluorescent intensity (MFI) of the samples which are analyzed by Flowjo software showed that by decreasing the rCTLA4-Ig concentrations, MFI levels logically reduced (Figure 9C). All of flow cytometry analyses were repeated three times with almost equal results. The average of values is shown in Figure 9. 

*Evaluation of T lymphocyte inhibition by rCTLA4-Ig*

**Figure 10:**
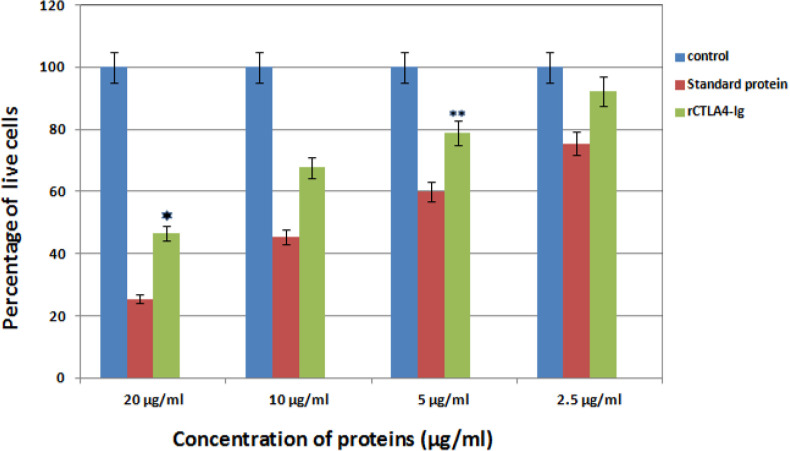
The bar graph represents Comparison inhibitory effect of standard protein and rCTLA4-Ig. Blue bars show control well in which was not used rCTLA4-Ig protein. so almost 100% cells stayed alive. Green bars depict inhibitory effect of rCTLA4-Ig on T cells. By increasing rCTLA4-Ig concentration, T cell proliferation decreased. The standard protein (red bars), which was bought from Adipogen company, had better function than rCTLA4-Ig in all concentrations. There was a significant difference between standard and rCTLA4-Ig. Value significantly different from standard protein only at *P < 0.05 or **P < 0.01

After treatment with different standard and rCTLA4-Ig concentrations, T cell survival was measured by MTT method. The results showed that rCTLA4-Ig could inhibit the proliferation of T lymphocytes under *in-vivo* conditions. In addition, by increasing the concentration of rCTLA4-Ig, the proliferation intensity decreased. It was also found out that inhibitory effect of rCTLA4-Ig was lower than standard protein. The treatment was repeated three times with almost similar quantities and the mean measurement is depicted in Figure 10.

**Table 1:** structural parts of designed chimeric protein**s**

**Chimeric proteins**	**Functional section**	**linker**	**Stabilization section**	**Substituting** ** in CTLA4 section (binding section)**
Abatacept	Extra domain of CTLA4	Q	FC of human IgG1	none
Belatacept	Extra domain of CTLA4	Q	FC of human IgG1	A29Y, L104E
structure 1	Extra domain of CTLA4	GGGGSGGGGSGGGGS	3DHSA	A29Y,L104E,K93Q,K28H
structure 2	Extra domain of CTLA4	AEAAAKEAAAKEAAAKA	3DHSA	A29Y,L104E,K93Q,K28H
structure 3	Extra domain of CTLA4	GGGGS	FC of human IgG1	A29Y,L104E,K93Q,K28H
structure 4	Extra domain of CTLA4	Q	FC of human IgG1	A29Y,L104E,K93Q,K28H
structure 5	Extra domain of CTLA4	Q	FC of human IgG1	A29Y,L104E,L58G,K28H
structure 6	Extra domain of CTLA4	Q	FC of human IgG1	A29Y,L104E,K93Q,L58G
structure 7	Extra domain of CTLA4	Q	FC of human IgG1	K93Q,K28H
structure 8	Extra domain of CTLA4	Q	FC of human IgG1	K93Q,A29H

**Table 2:** Physiological properties of the chimeric proteins using Expasy's Protparam

**Chimeric proteins**	**Number of amino acids**	**Molecular weight**	**Theoretical pI**	**The estimated half-life (mammalian reticulocytes** ***, in-vitro)***	**Instability index**	**Aliphatic index**	**Grand average of hydropathicity (GRAVY)**
Abatacept	357	39447.6	5.67	30 h	47.37	74.71	-0.403
Belatacept	357	39555.6	5.56	30 h	46.93	73.33	-0.432
structure 1	337	36855.0	5.33	30 h	40.30	77.18	-0.255
structure 2	339	37462.9	5.36	30 h	36.85	79.97	-0.240
structure 3	361	39809.9	5.42	30 h	47.61	72.52	-0.396
structure 4	357	39564.6	5.42	30 h	48.21	73.33	-0.429
structure 5	357	39508.5	5.53	30 h	48.16	72.24	-0.442
structure 6	357	39499.5	5.44	30 h	46.50	72.24	-0.443
structure 7	357	39456.5	5.52	30 h	47.43	74.71	-0.400
structure 8	357	39513.6	5.63	30 h	46.98	74.43	-0.416

**Table 3:** The secondary structure of chimeric proteins were predicted by GORIV

**Chimeric proteins**	**Sequence length**	**Alpha helix (Hh)**	**Extended strand**	**Random coil**
Abatacept	357	9.52%	32.77%	57.70%
Belatacept	357	7.28%	34.45%	58.26%
structure 1	337	21.96%	25.82%	52.23%
structure 2	339	28.32%	25.07%	46.61%
structure 3	361	7.20%	34.07%	58.73%
structure 4	357	7.28%	34.45%	58.26%
structure 5	357	7.28%	34.45%	58.26%
structure 6	357	7.28%	34.45%	58.26%
structure 7	357	9.52%	32.77%	57.70%
structure 8	357	7.28%	35.01%	57.70%

**Table 4:** he structural stability of the chimeric protein using Ramachandran plot

**Chimeric proteins**	**Number of residues in favored region**	**Number of residues in allowed region**	**Number of residues in outlier region**
Abatacept	300 (83.6%)	46 (12.8%)	13 (3.6%)
Belatacept	306 (86.2%)	37 (10.4%)	12 (3.4%)
structure 1	292 (87.2%)	33 (9.9%)	10 (3.0%)
structure 2	293 (86.9%)	28 (8.3%)	16 (4.7%)
structure 3	295 (82.2%)	42 (11.7%)	22 (6.1%)
structure 4	311 (87.6%)	31 (8.7%)	13 (3.7%)
structure 5	281 (79.2%)	51 (14.4%)	23 (6.5%)
structure 6	305 (85.9%)	38 (10.7%)	12 (3.4%)
structure 7	307 (86.5%)	36 (10.1%)	12 (3.4%)
structure 8	310 (87.3%)	32 (9.0%)	13 (3.7%)

**Table 5:** Thermodynamic details related to 5’ end of mRNAs were obtained by mfold tool

**Chimeric proteins**	**Minimum free energy**	**Structural Element**	**Initial ΔG**	**Number of initial loop base**
Abatacept	-336.10	Helix	-5.60	4 base pairs
Belatacept	-362.50	Helix	-5.50	3 base pairs
structure 1	-321.60	Helix	-4.60	3 base pairs.
structure 2	-333.40	Helix	-5.50	3 base pairs
structure 3	-377.30	Helix	-5.60	4 base pairs
structure 4	-328.50	Helix	-2.10	2 base pairs
structure 5	-354.30	Helix	-5.60	4 base pairs
structure 6	-334.10	Helix	-7.60	4 base pairs.
structure 7	-337.40	Helix	-3.40	2 base pairs.
structure 8	-353.40	Helix	-5.80	4 base pairs

**Table 6:** Affinity prediction of chimeric proteins using PPA-Pred server

**Chimeric proteins**	**Predicted value of Delta G ** **(binding free energy)**		**Predicted value of Kd** **(dissociation constant)**	
	**CD80**	**CD86**	**CD80**	**CD86**
Abatacept	-10.48 kcal/mol	-8.93 kcal/mol	2.06e-08 M	2.80e-07 M
Belatacept	-10.42 kcal/mol	-8.87 kcal/mol	2.29e-08 M	3.11e-07 M
structure1	-15.01 kcal/mol	-13.38kcal/mol	9.76e-12 M	1.55e-10 M
structure 2	-15.65 kcal/mol	-14.03kcal/mol	3.33e-12 M	5.14e-11 M
structure 3	-10.92 kcal/mol	-9.36 kcal/mol	9.85e-09 M	1.37e-07 M
structure 4	-10.48 kcal/mol	-8.94 kcal/mol	2.05e-08 M	2.79e-07 M
structure 5	-10.39 kcal/mol	-8.84 kcal/mol	2.41e-08 M	3.28e-07 M
structure 6	-10.47 kcal/mol	-8.92 kcal/mol	2.10e-08 M	2.86e-07 M
structure 7	-10.54 kcal/mol	-9.00 kcal/mol	1.85e-08 M	2.51e-07 M
structure 8	-10.42 kcal/mol	-8.88 kcal/mol	2.27e-08 M	3.09e-07 M

**Table 7:** Antigenicity index of chimeric proteins using vaxijen server

**Chimeric proteins**	**Overall Prediction for the Antigen**	**ANTIGEN/ NON-ANTIGEN**
Abatacept	0.4877	Probable NON-ANTIGEN
Belatacept	0.4819	Probable NON-ANTIGEN
structure 1	0.6558	Probable ANTIGEN
structure 2	0.6105	Probable ANTIGEN
structure 3	0.5099	Probable ANTIGEN
structure 4	0.4764	Probable NON-ANTIGEN
structure 5	0.4762	Probable NON-ANTIGEN
structure 6	0.4831	Probable NON-ANTIGEN
structure 7	0.4815	Probable NON-ANTIGEN
structure 8	0.4899	Probable NON-ANTIGEN

**Table 8:** Comparison of CD spectra results and predicted data of rCTLA4-Ig

**Secondary** **structure**	**Random coil (%)**	**Beta-sheet (%)**	**Alpha-helix (%)**	**Beta-turn**
Predicted	58.26%	34.45%	7.28%	Not calculated
CD	50.7%	31.3%	5.3%	12.7%

## Discussion

Some family members of CD28 receptor have inhibitory role on activated T cells. One of them is CTLA4 receptor that is expressed on activated T cells and binds to B7 molecules on APC to inhibit activated T cells (1, 28-30), because CTLA4 affinity to B7 ligands is 10 to 15 times stronger than CD28 (31). Abatacept, a Soluble fusion protein consists of the extracellular domain of CTLA4 and FC fragment of IgG molecule, was generated to prevent some autoimmune diseases especially Rheumatoid Arthritis (32-34). A more effective drug, called belatacept was manufactured by creating two substitutions (A29Y, L104E) in CTLA4 binding domain a few years later (8, 9, 34-36). 

In this study, a new model of CTLA4-Ig was designed by replacing some amino acids in CTLA4 fragment and altering FC section to 3DHSA in order to gain more efficient chimeric protein than belatacept. 

To achieve this goal, insilico techniques were used to predict different features of designed proteins such as 2D and 3D structures, physiochemical properties, stability, antigenicity index and affinity which leads to cheap and fast designing process (37, 38). According to bioinformatics results, the proper model was cloned and expressed in *E.coli (BL21DE3)* successfully, and rCTLA4-Ig was compared with predicted model. First of all, eight models were designed (Table1). In structure 1 and 2, FC fragment was replaced with 3DHSA. It is used as a fusion partner to increase circulating time that the short-acting drugs can survive in serum (longer than 19 days) (39-41). Short peptide was applied as the linker to separate two segments. A flexible linker ((GGGGS) 3) was used to enhance protein stability and proper folding in structure 1. A rigid linker (A (EAAAK) 3A) was applied in structure 2 in order to improve biological activity (42-44). Also, a flexible linker (GGGGS) used for improving biological activity in structure 3 (45). Seob, Sh. and his coworkers (2007) used a flexible peptide to express canine CTLA4-Ig and was able to produce proper amount of rCTLA4-Ig with biological activity (46). FC molecule was applied as a stabilization fragment in the third model. Belatacept sequence was used in models 4 to 8. Only a Q residue was added because the hinge sequences (12a.a) acted as a linker. According to 3D structure prediction results, inserted linkers could separate two segments of chimeric proteins efficiently, and it could inhibit unfavorable interactions between stabilization and functional domains. Zhenghai. Xu. and his partners (2012) surveyed effects of different point substitutions in the CTLA4-Ig binding regions.(10) Two new substitutions in the CTLA4 fragment of each structure were performed by exploiting their study. Designed structures were compared with belatacept as a standard protein structure. Chimeric protein properties were calculated using Protparam tool (Table2) (47). There was not any difference between the estimated values of the half-life (30 h) in any of the structures. The half-life is the prediction of how much time takes for half of a protein in cell to disappear after its synthesis in the cell. The instability index is a scale to estimate the stability of protein in a test tube. A protein in which instability index is smaller than 40 is predicted as stable, and if the instability index is more than 40, it is unstable. Therefore, all of structures are unstable except structure 2 (instability index: 36.85). The aliphatic index of a protein is defined as the relative volume occupied by aliphatic side chains (Alanine, Valine, Isoleucine, and Leucine). This can show thermo stability in globular proteins so structure 2 (aliphatic index: 79.97) has the highest, and structure 5 and 6 (aliphatic index: 72.24) have the lowest thermo stability. The gravy value for a peptide or protein is calculated as the sum of hydropathy values of all the amino acids, divided by the number of residues in the sequence. The lower gravy of proteins the better interaction with water it has and the structure 6 has the lowest gravy. According to gorIV prediction (Table 3) structures 4, 5, and 6 are completely similar to belatacept and consist of 7.28% alpha helix, 34.45% extended strand, and 58.26% random coil. Moreover, 3D structure prediction by I-Tasser server (Figure2) revealed huge changes in the structure 1 and 2 in comparison with standard drug structure and proposed that using 3DHSA as a stabilizer was not suitable. On the contrary, 3D structure prediction of the other structures showed that the predicted 3D structures follow similar features as in belatacept structure. C-score is typical in the range of [−5 to 2], where a c-score of higher value signifies the model with a high confidence. The highest c-score was related to structure 6 (c-score:0.58).In accordance with Ramachandran plot (Table 4), most residues of all chimeric proteins are in stable zone, which means more than 85% of residues have allowed conformation, and structure 6 and 7 are more similar to belatacept structure, in comparison with the other structures. In addition, ProSA server results demonstrated that structure 6 was inside a range characteristic for native proteins (Figure 3B). Verify3D analyses of structures also represented that almost 87.67% the amino acid residues of structure 6 have scored >= 0.2 in the 3D/1D profile. 

RNA secondary structure prediction was done by mfold tool, which distinguishes the potential folding of the chimeric gene. The 5’ terminus of the structure 6 gene folded in a typical way of all prokaryotic gene structures (Table 5). The data has shown that mRNA was stable enough for efficient translation in bacterial host. This fact was confirmed by high level expression of structure 6 in experimental efforts. The structures were surveyed by Zdock server to investigate whether functional domain of structure 6 bound correctly to B7 ligands or not (Figure5). The results showed the structure 6 bound to proper site of B7 ligands. According to data for affinity prediction of chimeric proteins by PPA-Pred server (Table 6), the strongest affinity is related to structure 2. This discrepancy could be explained by this evidence that the percentage of binding site residues plays an important role in governing protein-protein binding affinity (22, 23). Based on the indexes extracted from VaxiJen server (Table7), overall prediction of the antigen for structure 1 and 2 is probable antigen, because alternation in 2D and 3D structures of structure 1 and 2 exposed more conformational epitopes on protein surface as a result antigenicity index increased. 

Eventually based on the bioinformatic observations, structure 6 was selected due to its similarity to the second and third structures of belatacept structure, the best validation score, low antigenicity index, proper protein and mRNA stability, suitable affinity to CD80 and CD86 ligands. Structure 6 was translated back to nucleotide sequence. Then, codon usage and the overall GC content were optimized for expression in bacterial host. In addition, the ribosomal binding sites and the restriction enzymes sites within the sequence which might interfere with cloning were removed. Proper optimization led to high level rCTLA4-Ig expression in host (10µg/mL). According to our bioinformatics results, rCTLA4-Ig gene was cloned in the pET28a vector and after transformation, the recombinant vector was expressed in *E.coli (BL21DE3)*. In previous studies, CTLA4-Ig has often been expressed in eukaryotic cells. For example, Zang *et al*. and his partners (2007) cloned and expressed the linear plasmids containing the CTLA4-Ig gene in *CHO* cells. Expression of CTLA4-Ig in cell lines is linked with difficulties such as the complexity of the extraction process and the risk of viral contamination. On the contrary, expression in bacterial systems has some advantages such as rapid culturing, and lower cost of downstream processes (48). RCTLA4-Ig was purified under denature condition. The protein was refolded due to urea serial concentration. Efficiency of this purification protocol was remarkable and about 10 µg/mL protein was achieved. In comparison with the other studies such as, Seob, Sh. and his partner (2007), with protein yielding about 6 µg/mL, this is a valuable achievement. The secondary structure of predicted chimeric protein was compared with CD analysis to validate bioinformatics software’s data, and as shown in table 8, CD spectra analysis and predicted data are almost similar. Nevertheless, the beta turn structure was not predicted by bioinformatics analysis, the CD analysis showed 12% beta turn structure in rCTLA4-Ig.

ELISA analysis with Anti-human IgG antibody confirmed that rCTLA4-Ig has a proper structure. These findings proved that the predicted structure is affordable and is a proper model for future researches.

Flow cytometry technique was used to determine the affinity of rCTLA4-Ig to CD80 and CD86 receptors. Zangai *et al.* (2012) applied flow cytometry method to investigate the effect of different mutations on affinity of CTLA4-Ig fusion protein. The *Raji* cells express high level of B7 receptor on its surface. The expression of this receptor was confirmed by using an anti-CD80-PE antibody. Considering that the stabilization fragment of fusion protein contains the FC part of the human IgG1 antibody, an anit-human IgG-FITC antibody was used to evaluate the binding assay. The flow cytometry results showed that the mean fluorescence intensity (MFI) directly correlated with concentration of rCTLA4-Ig and standard protein. The MFI of rCTLA4-Ig in different concentrations is also lower than the standard protein. This difference may be related to this fact that the standard protein has been produced in *CHO* cells and the CTLA4 protein has glycosylated parts and disulfide bonds. These modifications may affect its affinity whereas, in the bacterial cells do not perform these modifications (49). Undeniably, glycosylation has affected many important biological processes at both cellular and protein level. Protein drugs already must be glycosylated properly to exhibit optimal therapeutic effectiveness (50, 51). Previous studies have been mentioned that the most immune receptors are regulated by N- and O- glycosylation. CTLA4 also consist of multiple N- and O-glycosylation sites which have been characterized to modulate its maintenance at T cell surface and therefore its affinity for CD80/CD28 on APCs. Accordingly, a Thr 17 Ala substitution in human CTLA-4 resulted in decrease of N-glycosylation sites, which limited CTLA-4 stability at T cell surface (52, 53). According to previous studies, removing the glycosylation site of CTLA4 (N78) resulted in reduction of its binding affinity. This is proposed that glycosylation is vital for preserving the monomeric status of CTLA4 (54, 55).

Splenocytes of sensitive mice were utilized to determine inhibitory effect of rCTLA4-Ig. Lin.,Wan and coworkers (2010) also obtained splenocytes from balb/c mice to investigate the immunosuppressive activity of the purified LEA29Y (56). The results of inhibitory effect of rCTLA4-Ig on T lymphocytes showed that rCTLA4-Ig could inhibit proliferation of T lymphocytes under in-vivo conditions. The percentage of live lymphocyte cells were decreased by increasing concentration of rCTLA4-Ig. In addition, the inhibitory effect of rCTLA4-Ig was also compared with the standard protein. It was demonstrated that inhibitory effect for rCTLA4-Ig was weaker than the standard protein. This is due to the weaker Affinity of rCTLA4-Ig to its receptor.

## References

[1] Walunas TL, Lenschow DJ, Bakker CY, Linsley PS, Freeman GJ, Green JM, Thompson CB, Bluestone JA (1994). CTLA-4 can function as a negative regulator of T cell activation. Immunity..

[2] Vaughan AN, Malde P, Rogers NJ, Jackson IM, Lechler RI, Dorling A (2000). Porcine CTLA4-Ig lacks a MYPPPY motif, binds inefficiently to human B7 and specifically suppresses human CD4+ T cell responses costimulated by pig but not human B7. J. Immuno..

[3] Walker LS, Sansom DM (2011). The emerging role of CTLA4 as a cell-extrinsic regulator of T cell responses. Nat. Rev. Immunol..

[4] Mccoy KD, Le gros G (1999). The role of CTLA-4 in the regulation of T cell immune responses. Immunol. Cell Biol..

[5] Karandikar NJ, Vanderlugt CL, Walunas TL, Miller SD, Bluestone JA (1996). CTLA-4: a negative regulator of autoimmune disease. J. Exp. Med..

[6] Mola EM, Balsa A, Taboada VM, Sanmartí R, Marenco JL, Sarabia FN, Gómez J, Álvaro J M, Ivorra JA, Lojo L (2013). Abatacept use in rheumatoid arthritis: evidence review and recommendations. Reumatol. Clín..

[7] Cross AH, Girard T, Giacoletto K, Evans R, Keeling R, Lin R, Trotter R (1995). Long-term inhibition of murine experimental autoimmune encephalomyelitis using CTLA-4-Fc supports a key role for CD28 costimulation. J. Clin. Investig..

[8] Larsen CP, Pearson TC, Adams AB, Tso P, Shirasugi N, Strobert ME, Anderson D, Cowan S, Price K, Naemura J (2005). Rational development of LEA29Y (belatacept), a high-affinity variant of CTLA4-Ig with potent immunosuppressive properties. Am. J. Transplant.

[9] Vincenti F, Larsen C, Durrbach A, Wekerle T, Nashan B, Blancho G, Lang P, Grinyo J, Halloran PF, Solez K (2005). Costimulation blockade with belatacept in renal transplantation. N. Engl. J. Med..

[10] Xu Z, Juan V, Ivanov A, Ma Z, Polakoff D, Powers DB, DuBridge RB, Wilson K, Akamatsu Y (2012). Affinity and cross-reactivity engineering of CTLA4-Ig to modulate T cell costimulation. J. Immunol..

[11] Peraino JS, Zhang H, Li G, Huang CA, Wang Z (2013). Molecular basis of cross-species reactivities of human versus porcine CTLA-4. Hum. immunol..

[12] Gurpreet K, Pratap KP (2018). In silico physicochemical characterization and topology analysis of Respiratory burst oxidase homolog (Rboh) proteins from Arabidopsis and rice. Bioinformatics.

[13] Zuker M (2003). Mfold web server for nucleic acid folding and hybridization prediction. Nucleic Acids Res..

[14] Gruber AR, Lorenz R, Bernhart SH, Neuböck R, Hofacker IL (2008). The vienna RNA websuite. Nucleic Acids Res..

[15] Garnier J, Gibrat JF, Robson B (1996). GOR method for predicting protein secondary structure from amino acid sequence. Methods Enzymol..

[16] Xia F, Dou Y, Lei G, Tan Y (2011). FPGA accelerator for protein secondary structure prediction based on the GOR algorithm. BMC Bioinform..

[17] Roy A, Kucukural A, Zhang Y (2010). I-TASSER: a unified platform for automated protein structure and function prediction. Nat. protocol..

[18] Zhang Y (2008). I-TASSER server for protein 3D structure prediction. BMC Bioinform..

[19] Roy A, Yang J, Zhang Y (2012). COFACTOR: an accurate comparative algorithm for structure-based protein function annotation. Nucleic Acids Res..

[20] Lovell SC, Davis IW, Arendall WB, Bakker PI, Word JM, Prisant MG, Richardson JS, Richardson DC (2003). Structure validation by Cα geometry: ϕ, ψ and Cβ deviation. Proteins.

[21] Ritchie DW (2008). Recent progress and future directions in protein-protein docking. Curr. Protein Pept. Sci..

[22] Pierce BG, Hourai Y, Weng Z (2011). Accelerating protein docking in ZDOCK using an advanced 3D convolution library. PloS one.

[23] Yugandhar K, Gromiha MM (2014). Protein-protein binding affinity prediction from amino acid sequence. Bioinformatics.

[24] Yugandhar K, Gromiha MM (2015). Response to the comment on ‘protein-protein binding affinity prediction from amino acid sequence’. Bioinformatics.

[25] Doytchinova IA, Flower DR (2007). VaxiJen: a server for prediction of protective antigens, tumour antigens and subunit vaccines. BMC Bioinform..

[26] Doytchinova IA, Flower DR (2007). Identifying candidate subunit vaccines using an alignment-independent method based on principal amino acid properties. Vaccine.

[27] Kruger NJ (2009). The Bradford method for protein quantitation. The protein protocols handbook.

[28] Teft WA, Kirchhof MG, Madrenas J (2006). A molecular perspective of CTLA-4 function. Annu. Rev. Immunol..

[29] Qureshi OS, Zheng Y, Nakamura K, Attridge K, Manzotti C, Schmidt EM, Baker J, Jeffery LE, Kaur S, Briggs Z (2011). Trans-endocytosis of CD80 and CD86: a molecular basis for the cell-extrinsic function of CTLA-4. Science.

[30] Ruperto N, Lovell DJ, Quartier P, Paz E, Rubiopérez N, Silva CA, Abudmendoza C, burgosvargas R, Gerloni V, Melogomes JA (2008). Abatacept in children with juvenile idiopathic arthritis: a randomised, double-blind, placebo-controlled withdrawal trial. Lancet.

[31] O’Rourke RW, Kang SM, Lower JA, Feng S, Ascher NL, Baekkeskov S, Stock PG (2000). A denderitic cell line genetically modified to express CTLA4-Ig as a means to prolong islet allograft survival. Transplantation.

[32] Khraishi MM (2014). Experience with subcutaneous abatacept for rheumatoid arthritis: an update for clinicians. Ther. Adv. Musculoskel. Dis..

[33] Genovese MC, Becker JC, Schiff M, Luggen M, Sherrer Y, Kremer J, Birbara C, Box J, Natarajan K, Nuamah I (2005). Abatacept for rheumatoid arthritis refractory to tumor necrosis factor α inhibition. N. Engl. J. Med..

[34] Kremer JM, Genant HK, Moreland LW, Russell AS, Emery P, Abudmendoza C, Szechinski J, Li T, Gezandbecker JC (2006). Effects of abatacept in patients with methotrexate-resistant active rheumatoid arthritis: a randomized trial. Ann. Intern. Med..

[35] Vincenti F, Blancho G, Durrbach A, Friend P, Grinyo J, Halloran PF, Klempnauer J, Lang P, Larsen CP, Mühlbacher F (2010). Five-year safety and efficacy of belatacept in renal transplantation. J. Am. Soc. Nephrol..

[36] Vincenti F, Rostaing L, Grinyo J, Rice K, Steinberg S, Gaite L, Moal MC, Mondragon GA, Kothari J, Polinsky MS (2016). Belatacept and long-term outcomes in kidney transplantation. N. Engl. J. Med..

[37] Macdonald JT, Barnes C, Kitney RI, Freemont PS, Stan GBV (2011). Computational design approaches and tools for synthetic biology. Integr. Biol..

[38] Wang Y, Xing J, Xu Y, Zhou N, Peng J, Xiong Z, Liu X, Luo X, Luo C, Chen K (2015). In silico ADME/T modelling for rational drug design. Q. R. Biophysics.

[39] Sheffield WP, Eltringhamsmith LJ (2011). Incorporation of albumin fusion proteins into fibrin clots in-vitro and in-vivo: comparison of different fusion motifs recognized by factor XIIIa. BMC Biotechnol..

[40] Zhao S, Zhang Y, Tian H, Chen X, Cai D, Yaowand X (2013). Extending the serum half-life of G-CSF via fusion with the domain III of human serum albumin. BioMed Res. Int..

[41] Kenanova VE, Olafsen T, Salazar FB, Williams LE, Knowles S, Wu AM (2010). Tuning the serum persistence of human serum albumin domain III: diabody fusion proteins. Pro. Eng..

[42] Chen X, Zaro JL, Shen WC (2013). Fusion protein linkers: property, design and functionality. Adv. Drug Deliv. Rev..

[43] George RA, Heringa J (2002). An analysis of protein domain linkers: their classification and role in protein folding. Pro. Eng..

[44] Wriggers W, Chakravarty S, Jennings PA (2005). Control of protein functional dynamics by peptide linkers. Pept. Sci..

[45] Arai R, Ueda H, Kitayama A, Kamiya N, Nagamune T (2001). Design of the linkers which effectively separate domains of a bifunctional fusion protein. Pro. Eng..

[46] Shin IS, Choi EW, Chung JY, Hwang CY, Lee CW, Youn HY (2007). Cloning, expression and bioassay of canine CTLA4Ig. Vet. Immunol..

[47] Gasteiger E, Hoogland C, Gattiker A, Wilkins MR, Appel RD, Bairoch A (2005). Protein identification and analysis tools on the ExPASy server. The proteomics protocols handbook.

[48] Demain AL, Vaishnav P (2009). Production of recombinant proteins by microbes and higher organisms. Biotechnol. Adv..

[49] Zhu L, Guo Q, Guo H, Liu T, Zheng Y, Gu P, Chen X, Wang H, Hou Y (2014). Versatile characterization of glycosylation modification in CTLA4-Ig fusion proteins by liquid chromatography-mass spectrometry. MAbs..

[50] Solá RJ, Griebenow K (2009). Effects of glycosylation on the stability of protein pharmaceuticals. J. Pharm. Sci..

[51] Xiong Y, Karuppanan K, Bernardi A, Li Q, Kommineni V, Lebrilla CB, Dandekar AM, Faller R, McDonald KA, Nandi S (2019). Effects of N-glycosylation on the structure, function, and stability of a plant-made Fc-fusion anthrax decoy protein. Fron. Plant Sci..

[52] Mkhikian H, Grigorian A, Li CF, Chen HL, Newton B, Zhou RW, Beeton C, Torossian S, Tatarian GG, Lee SU (2011). Genetics and the environment converge to dysregulate N-glycosylation in multiple sclerosis. Nat. Commun..

[53] Pereira MS, Alves I, Vicente M, Campar A, Silva MC, Padrão NA, Pinto V, Fernandes Â, Dias AM, Pinho SS (2018). Glycans as Key Checkpoints of T Cell Activity and Function. Fron. Immunol..

[54] Hufton SE, Neer N, Beuken T, Desmet J, Sablon E, Hoogenboom HR (2000). Development and application of cytotoxic T lymphocyte-associated antigen 4 as a protein scaffold for the generation of novel binding ligands. FEBS Lett..

[55] Darlington PJ, Kirchhof MG, Criado G, Sondhi J, Madrenas J (2005). Hierarchical regulation of CTLA-4 dimer-based lattice formation and its biological relevance for T cell inactivation. J. Immunol..

[56] Wan L, Zhu S, Li Y, Liu S, Yang H, Li S, Li Y, Cheng J, Lu X (2011). Production and characterization of LEA29Y, a variant of cytotoxic T-lymphocyte antigen 4-immunoglobulin, in Pichia pastoris. Appl. Microbiol. Biotechnol..

